# Uncovering the complex genetic architecture of human plasma lipidome using machine learning methods

**DOI:** 10.1038/s41598-023-30168-z

**Published:** 2023-02-22

**Authors:** Miikael Lehtimäki, Binisha H. Mishra, Coral Del-Val, Leo-Pekka Lyytikäinen, Mika Kähönen, C. Robert Cloninger, Olli T. Raitakari, Reijo Laaksonen, Igor Zwir, Terho Lehtimäki, Pashupati P. Mishra

**Affiliations:** 1 Department of Clinical Chemistry, Faculty of Medicine and Health Technology, Tampere University, Tampere, Finland; 2 Faculty of Medicine and Health Technology, Finnish Cardiovascular Research Center Tampere, Tampere University, Tampere, Finland; 3 Department of Clinical Chemistry, Fimlab Laboratories, Tampere, Finland; 4 Department of Computer Science and Artificial Intelligence, Andalusian Research Institute in Data Science and Computational Intelligence (DaSCI), University of Granada, Granada, Spain; 5 Department of Clinical Physiology, Tampere University Hospital, Tampere, Finland; 6 Department of Psychiatry, Washington University School of Medicine, St. Louis, MO USA; 7 Research Centre of Applied and Preventive Cardiovascular Medicine, University of Turku, Turku, Finland; 8 Department of Clinical Physiology and Nuclear Medicine, Turku University Hospital, Turku, Finland; 9 Centre for Population Health Research, University of Turku and Turku University Hospital, Turku, Finland; 10 Zora Biosciences Oy, Espoo, Finland; 11 Instituto de Investigación Biosanitaria ibs. GRANADA, Complejo Hospitales Universitarios de Granada/Universidad de Granada, Granada, Spain

**Keywords:** Computational biology and bioinformatics, Genetics, Molecular biology

## Abstract

Genetic architecture of plasma lipidome provides insights into regulation of lipid metabolism and related diseases. We applied an unsupervised machine learning method, PGMRA, to discover phenotype-genotype many-to-many relations between genotype and plasma lipidome (phenotype) in order to identify the genetic architecture of plasma lipidome profiled from 1,426 Finnish individuals aged 30–45 years. PGMRA involves biclustering genotype and lipidome data independently followed by their inter-domain integration based on hypergeometric tests of the number of shared individuals. Pathway enrichment analysis was performed on the SNP sets to identify their associated biological processes. We identified 93 statistically significant (hypergeometric *p*-value < 0.01) lipidome-genotype relations. Genotype biclusters in these 93 relations contained 5977 SNPs across 3164 genes. Twenty nine of the 93 relations contained genotype biclusters with more than 50% unique SNPs and participants, thus representing most distinct subgroups. We identified 30 significantly enriched biological processes among the SNPs involved in 21 of these 29 most distinct genotype-lipidome subgroups through which the identified genetic variants can influence and regulate plasma lipid related metabolism and profiles. This study identified 29 distinct genotype-lipidome subgroups in the studied Finnish population that may have distinct disease trajectories and therefore could be useful in precision medicine research.

## Introduction

Atherosclerosis, the underlying pathology behind many cardiovascular diseases (CVDs), is a heterogeneous lipid accumulation and inflammation related disease with roots including genetics^1^, personality^2^, and lifestyle factors^3^. Previous lipidomic analyses have revealed several ceramides and phospholipids associated with key atherosclerosis processes such as uptake and aggregation of lipoproteins, accumulation of cholesterol within macrophages, production of superoxide anions, expression of cytokines and inflammation^4–6^. Similarly, genetic studies of traditional lipids such as total cholesterol (TC), HDL-cholesterol (HDL-C), LDL-cholesterol (LDL-C), non-HDL-cholesterol and triglycerides have identified about 1000 genomic loci and improved our understanding of lipid metabolism^7–10^. Some studies have reported genetic associations for subsets of lipidome^11–13^ and metabolome^13–20^. Only few genome-wide association studies (GWASs) of lipidome involving 141–596 lipid species have been done^21–23^. Therefore, genetic regulation of detailed lipidome beyond the traditional lipids is largely unknown.

While GWASs of traditional clinical lipids or lipidome using traditional linear or logistic regression model can identify loci associated with lipids across the whole studied population (global associations), they disregard potential subgroups within the studied population and their associations with lipids (local associations). Understanding of local associations is crucial for precision medicine because specific lipidome-based subgroups within a population may have different trajectories of disease development and may have varying disease or metabolic outcomes. Study of the complex genetics of the lipidome at the subgroups level within a population requires an alternative machine-learning-based bioinformatics approach, which is clearly lacking in the existing literature.

Therefore, in this study, our goal was to identify subgroups in the Young Finns Study (YFS) cohort participants with a distinct profile of sets of lipid species regulated by distinct sets of genetic variants using an alternative unsupervised machine learning approach. The machine learning approach referred as *phenotype-genotype many-to-many relation analysis* (PGMRA), involves a multilayer non-negative matrix factorization^24^ of genotype and phenotype (lipidome in this study) data and identification of biclusters separately^25–28^. Biclustering is simultaneous clustering of rows and columns of a matrix. For example, in case of lipidome data with samples on columns and molecular lipids on rows, a bicluster is a subset of the lipidome data matrix that contains subset of samples (columns) with similar profile across a subset of molecular lipids (rows). The identified biclusters (subgroups) in the genotype data are then associated with lipidome biclusters by testing the number of shared individuals between these biclusters and thus pinpointing significant relations. The overlap of individuals among the biclusters are tested using hypergeometric test. The bicluster pairs between the two data types are referred as *many-to-many relations*, which are complex in the sense that the same genotype may be associated with different lipid profiles (which is called multi-finality) and different genotypes may have the same lipid profile (which is called equifinality).

## Methods

### Study participants

This study was based on the Cardiovascular Risk in Young Finns Study (YFS), an ongoing Finnish longitudinal general population study on the evolution of cardiovascular risk factors from childhood to adulthood^29^. The study began in 1980 with 3,596 participants including children and adolescents aged 3–18 years, randomly selected from five university hospital catchment areas in Finland. The study was approved by the ethical committee of the Hospital District of Southwest Finland on 20 June 2017 (ETMK:68/1801/2017). All participants gave their written informed consent, and the studies were conducted in accordance with the Declaration of Helsinki. Data protection will be handled according to current regulations. The present study is based on 1,426 participants, aged 30–45, from the 2007 follow-up for whom genotype, plasma lipidome and covariate data were available. Characteristics of the study participants is summarized in Table 1.

**Table 1:** Population characteristics of the Cardiovascular Risk in Young Finns Study cohort. Data are expressed as mean ± SD or percentages.

	**Men**	**Women**
Number of subjects, N (%)	666 (47%)	760 (53%)
Age, years	38 ± 5	38 ± 5
Body mass index, kg/m^2^	26.8 ± 4.2	25.4 ± 5.1
Total cholesterol (mmol/l)	5.2 ± 0.9	4.9 ± 0.8
LDL cholesterol (mmol/l)	3.3 ± 0.8	3.0 ± 0.7
HDL cholesterol (mmol/l)	1.2 ± 0.3	1.4 ± 0.3
Triglycerides (mmol/l)	1.7 ± 1.2	1.2 ± 0.6
Serum glucose (mmol/l)	5.5 ± 0.8	5.2 ± 0.7
Insulin (IU/l)	9.7 ± 9.6	8.6 ± 7.6
C-reactive protein (mg/l)	1.6 ± 4.9	1.9 ± 3.2
Systolic blood pressure (mmHg)	125 ± 13	116 ± 14
Diastolic blood pressure (mmHg)	78 ± 11	73 ± 11
Alcohol consumption, units/day	1.4 ± 2	0.5 ± 0.7
Physical activity index (MET h/wk)	19 ± 22	19 ± 20
Daily smoking, %	111/575 (19%)	85/669 (13%)
Family risk factor for coronary heart disease (%)	87/577 (15%)	116/670 (17%)

### Genotyping and quality control

Genomic DNA was extracted from peripheral blood leukocytes from whole blood samples of YFS using a commercially available kit and Qiagen BioRobot M48 Workstation according to the manufacturer’s instructions (Qiagen, Hilden, Germany)^30^. Genotyping was performed at the Welcome Trust Sanger Institute using a custom-made Illumina Human 670 k BeadChips. Genotypes were determined using the Illuminus clustering algorithm. Fifty-six samples failed the Sanger genotyping pipeline quality control (QC) criteria (i.e. duplicated samples, heterozygosity, low call rate, or Sequenom fingerprint discrepancies)^30^. Three samples were removed due to a low genotyping call rate (< 0.95) and 54 samples were excluded for possible relatedness (pi.hat > 0.2). A total of 11,766 single SNPs were excluded based on the variation from Hardy–Weinberg equilibrium (HWE) test (*p* ≤ 1.0 × 10^−6^), 7,746 SNPs failed the missingness test (call rate < 0.95) and 34,596 SNPs failed the frequency test (MAF < 0.01). After quality control there were 2,443 samples and 546,677 genotyped SNPs available for further analysis^30^. However, only 1,426 of the 2,443 participants had complete data on lipidome and covariates from the 2007 follow-up and therefore were further analyzed [Supplementary Figures S1 and S2].

### Plasma lipidome profiling

Lipidome quantification for the stored serum samples was performed at Zora Biosciences Oy (Espoo, Finland). Lipid extraction was based on a previously described method^31^. In brief, 10 μl of 10 mM 2,6-di-tert-butyl-4-methylphenol (BHT) in methanol was added to 10 μl of the sample, followed by 20 μl of internal standards (Avanti Polar Lipids Inc., Alabaster, AL) and 300 μl of chloroform:methanol (2:1, v:v) (Sigma-Aldrich GmbH, Steinheim, Germany). The samples were mixed and sonicated in a water bath for 10 min, followed by a 40-min incubation and centrifugation (15 min at 5700 × *g*). The upper phase was transferred and evaporated under nitrogen. Extracted lipids were resuspended in 100 μl of water-saturated butanol and sonicated in a water bath for 5 min. Then, 100 μl of methanol was added to the samples before the extracts were centrifuged for 5 min at 3500 × *g*, and finally the supernatants were transferred to the analysis plate for mass spectrometric (MS) analysis. The MS analyses have also been described in detail previously^32^. The analyses were performed on a hybrid triple quadrupole/linear ion trap mass spectrometer (QTRAP 5500, AB Sciex, Concord, Canada) equipped with ultra-high-performance liquid chromatography (UHPLC) (Nexera-X2, Shimadzu, Kyoto, Japan). Chromatographic separation of the lipidomic screening platform was performed on an Acquity BEH C18, 2.1 × 50 mm id. 1.7 μm column (Waters Corporation, Milford, MA, USA). The data were collected using a scheduled multiple reaction monitoring algorithm and processed using Analyst and MultiQuant 3.0 software (AB Sciex). The heights of the peaks obtained from the MS analysis were normalized with the internal standard amount and sample volume. The details on the chromatography and mass spectrometry conditions have been previously described in^32^. Lipid profiles of all the 437 molecular lipid species in the lipidome were available for more than 99% of the participants and therefore included in the final analysis. The list of studied 437 lipids and their annotations are shown in Supplementary Table 1S.

### GWAS of human plasma lipidome

PGMRA with a big genetic data is computationally challenging. Therefore, in order to pre-select relevant SNPs for PGMRA, we performed genome-wide association (GWA) analysis between 546,677 genotyped SNPs and 437 lipid species of human plasma lipidome using PLINK v1.90^33,34^. The analysis was adjusted for sex, age, body mass index (BMI), type 2 diabetes, lipid medication and the first 10 genetic principal components (PC1-10) as covariates.

### PGMRA of genotype and lipidome data

**Figure 1:**
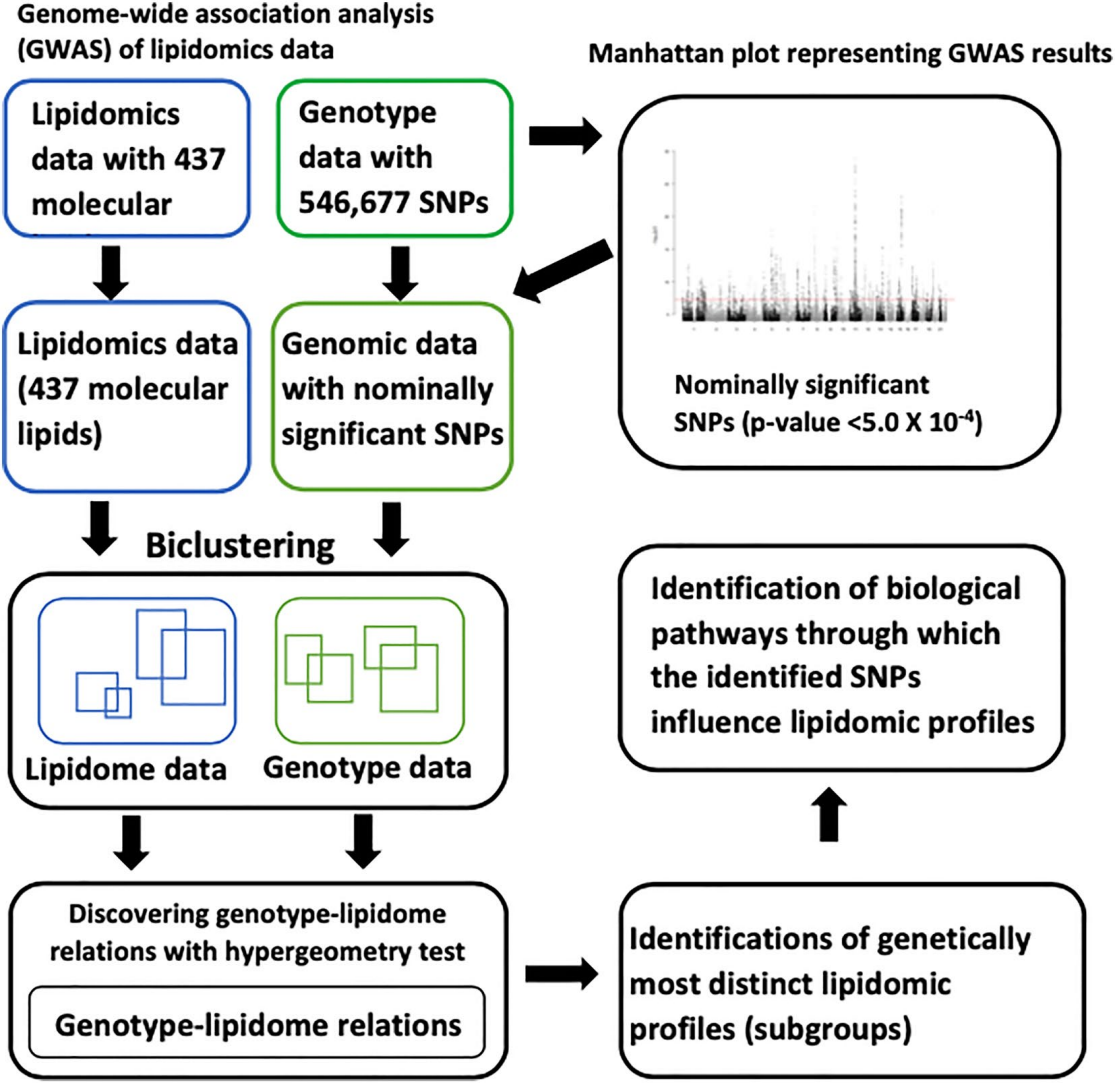
Phenotype-genotype many-to-many relation analysis (PGMRA) outline.

The PGMRA analysis was performed on the whole lipidomic data and subset of genotype data with SNPs that obtained nominal significance (*p*-value < 0.0005) in the GWA analysis of lipidome data as described elsewhere^25–28^. We implemented liberal criteria of *p*-value < 0.0005 to preselect the SNPs to be maximally inclusive for the PGMRA analysis. Also, we optimized the threshold to be specifically *p*-value < 0.0005 to limit the number of preselected SNPs to maximum of 20,000. The analysis involved biclustering of both lipidomic (participants-by-lipids matrix) and genotype data (participants-by-SNPs matrix) separately using nonnegative matrix factorization (NMF) (Fig. 1)^25^. Biclustering with fuzzy NMF was implemented in order to allow a SNP/lipid or a participant to belong to more than one bicluster. Many-to-many relations between genotype and lipidome biclusters were identified by calculating the pairwise probability of intersection of participants between the biclusters using hypergeometric statistics^35^. We performed linkage disequilibrium (LD)-based pruning of the SNPs in biclusters of significant relations to identify independent SNPs before further biological process or pathway enrichment analysis using pairwise correlation method implemented in PLINK v1.90 with default pairwise r^2^ threshold of 0.5.

### Annotation of SNPs and pathway enrichment analysis

Annotation of the discovered SNPs were done using ensembl Variant Effect Predictor (VEP) and ensemble assembly GRCh37^36^. Pathway analysis of the corresponding list of genes was performed using overrepresentation analysis method implemented in the *clusterProfiler* R package^37^. The analysis was done against the gene sets representing biological processes from Gene Ontology database^38^ as well as gene sets representing biological pathways from Kyoto Encyclopedia of Genes and Genomes (KEGG) database^39^.

### Ethical approval

Informed consent was acquired from all the YFS participants, and the study was conducted according to the principles of Helsinki declaration. The YFS was approved by the ethical committee of the Hospital District of Southwest Finland on 20 June 2017 (ETMK:68/1801/2017). Data protection will be handled according to current regulations.

## Results

### Study population characteristics

The characteristics of the study population are shown in Table 1.

### GWAS of human plasma lipidome

**Figure 2:**
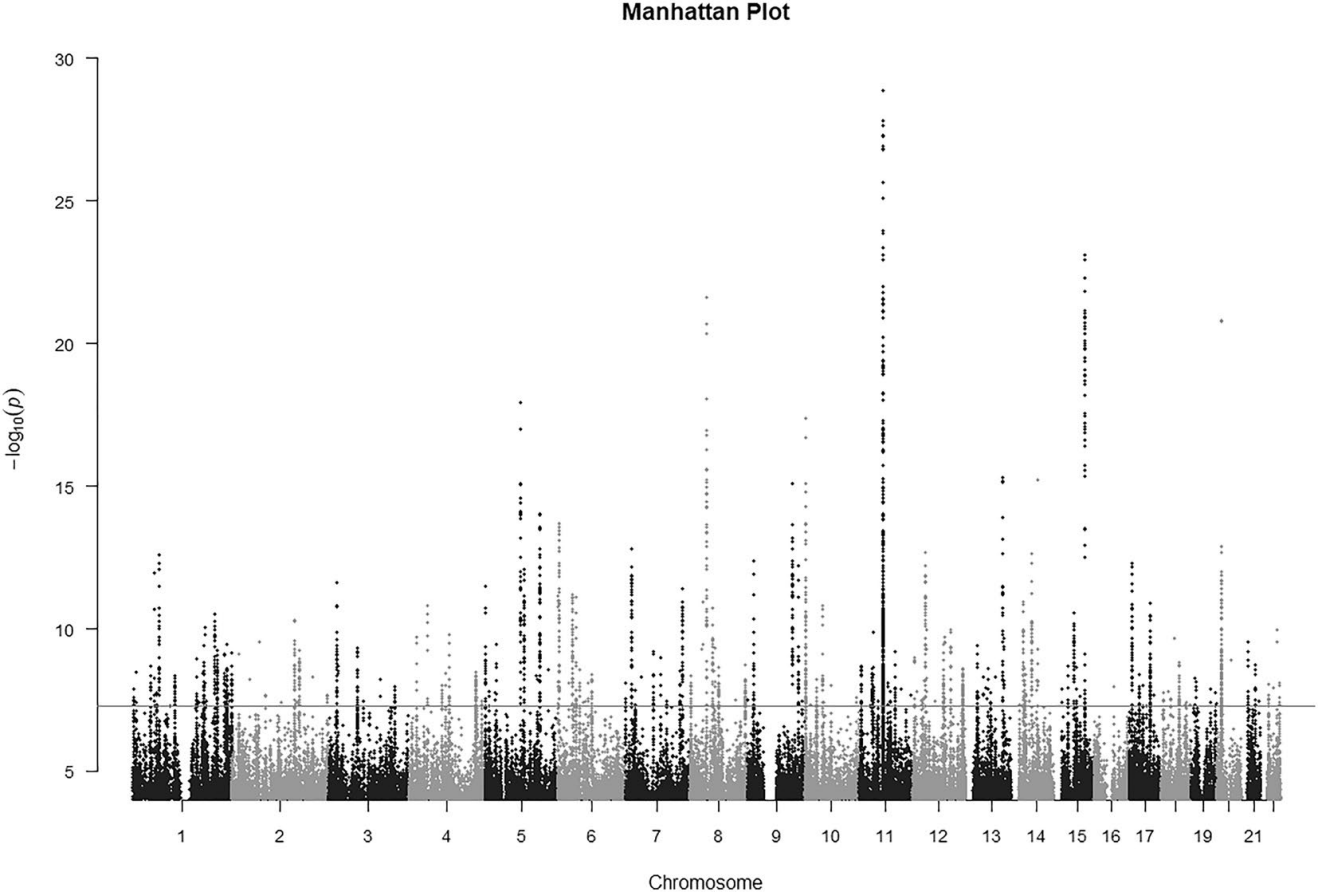
Manhattan plot showing results from GWAS of 437 lipid species results. The plot represents only the SNPs with nominal statistical significance (*p*-value < 5 × 10^−4^). SNPs above the red line represents those with *p*-value < 5 × 10^−8^.

**Figure 3:**
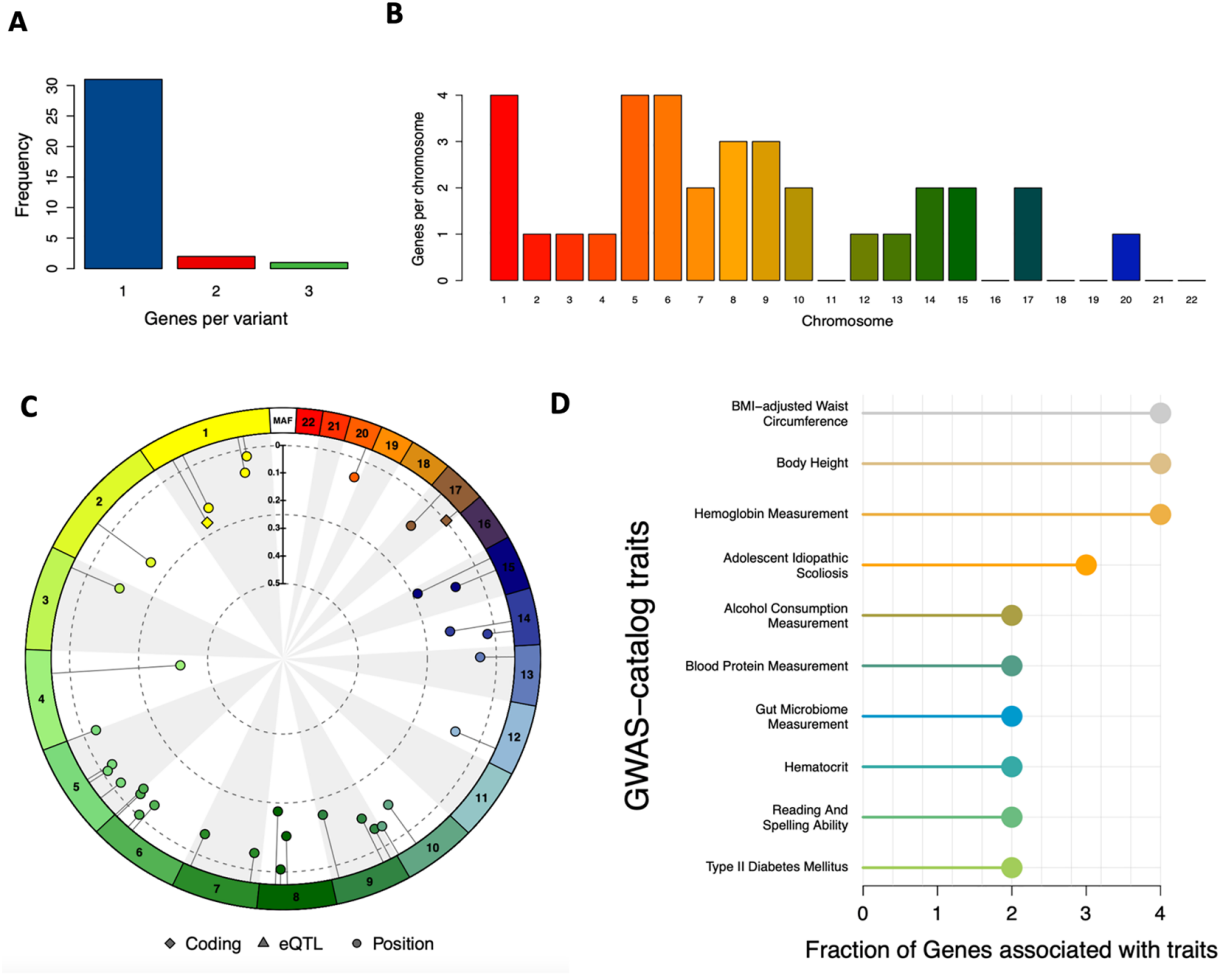
Results of the functional annotation of the 34 independent SNPs (single nucleotide polymorphisms) associated with different lipid species of human plasma lipidome.(**A**) Number of genes associated with each of the 34 independent SNPs.(**B**) Chromosomal distribution of all the 34 SNPs. (**C**) Circular summary figure showing the type of annotation of each SNP (coding, eQTL or annotated by their positions) as well as each SNP's minor allele frequency and chromosomal distribution.(**D**) Number of genes associated with the 34 independent SNPs (single nucleotide polymorphisms), expressed as fraction, for which a previous association was reported in the GWAS-catalog version 1.0.2 downloaded from https://www.ebi.ac.uk/gwas/docs/file-downloads.

GWAS of the 437 lipid species resulted into 51,707 SNP-lipid associations with nominal statistical significance (*p*-value < 0.0005) (Fig. 2) with 18,370 unique SNPs. There were 2340 SNP-lipid associations that were statistically significant at genome-wide level with *p*-value < 5 × 10^−8^ [Supplementary Table 2S] and 638 with study-wide significance levels with *p*-value < 1.1 × 10^−10^ [Supplementary Table 3S]. There were 65 unique SNPs in the 638 SNP-lipid associations, 21 of which have been reported by recent GWASs of human lipidome^21,23^. We identified 34 independent SNPs out of the 44 newly reported SNPs in the current study using PLINK based clumping with the r^2^ threshold of 0.1 and clumping window size of 250 kilo bases (Table 2). For interpreting the GWAS results, we prefer clumping to pruning as our goal is to select the most statistically significant SNP per region of LD. Pruning removes one SNP from the correlated pair of SNPs, keeping the one with the largest minor allele frequency, thus possibly removing the SNP with higher statistical significance. The 34 independent SNPs from the clumps were further analyzed using SnpXplorer^40^. SnpXplorer identified 38 genes associated with the 34 SNPs (Fig. 3A,B). Type of annotation of each of the SNPs (coding, eQTL or annotated by their positions) as well as their minor allele frequency and chromosomal distribution have been summarized in Fig. 3C. The genes associated with the SNPs have been reported by earlier GWASs to be associated with traits such as BMI-adjusted waist circumference, body height, type II diabetes mellitus, alcohol consumption and hemoglobin measurement (GWAS-catalog version 1.0.2 downloaded from https://www.ebi.ac.uk/gwas/docs/file-downloads) (Fig. 3D). However, no biological processes or pathways were identified to be enriched in the list of associated genes.

**Table 2:** Traditional GWAS for 437 plasma lipid species.

SNP	Position	Gene	Consequence	Lipid species	BETA	SE	*p*-value
rs925272	chr15:81,219,849	IL16	intron	TAG.14.0.18.2.18.2	2.83	0.27	8.2 × 10^−24^
rs7012713	chr8:42,737,160	CHRNB3	3_prime_UTR	TAG.18.2.18.2.18.2	2.6	0.26	2.5 × 10^−22^
rs157237	chr5:90,093,880	CTD-2151A2.3	intergenic	TAG.14.0.18.2.18.2	2.17	0.24	1.2 × 10^−18^
rs11252236	chr10:3,980,862	RP11-433J20.2	intergenic	TAG.18.2.18.2.18.2	2.09	0.23	4.0 × 10^−18^
rs9583985	chr13:91,998,895	GPC5	intron	TAG.18.2.18.2.20.4	3.77	0.45	5.0 × 10^−16^
rs7033785	chr9:107,393,025	LINC01509	intergenic	TAG.16.1.16.1.16.1	2.96	0.36	8.2 × 10^−16^
rs17170751	chr5:136,961,542	PRELID2	intergenic	TAG.16.1.16.1.16.1	2.85	0.36	9.7 × 10^−15^
rs9405270	chr6:5,460,429	FARS2	intron	TAG.15.0.16.0.18.1	1.35	0.17	2.1 × 10^−14^
rs10258334	chr7:15,731,926	RPL36AP26	intergenic	TAG.18.2.18.2.18.2	1.71	0.23	1.6 × 10^−13^
rs12317948	chr12:31,731,898	AMN1	upstream	TAG.18.2.18.2.18.2	3.16	0.42	2.1 × 10^−13^
rs2065079	chr14:50,784,059	NIN	intron	TAG.18.2.18.2.18.2	3.18	0.43	2.4 × 10^−13^
rs12030788	chr1:66,302,446	PDE4B	intron	TAG.14.0.18.2.18.2	3.28	0.44	2.5 × 10^−13^
rs3905248	chr9:15,297,030	TTC39B	intron	TAG.18.2.18.2.18.2	1.51	0.2	4.0 × 10^−13^
rs11653054	chr17:7,101,608	ASGR2	synonymous	TAG.15.0.16.0.18.1	1.98	0.27	5.0 × 10^−13^
rs10986211	chr9:124,100,867	LHX2	intergenic	TAG.18.2.18.2.20.4	2.68	0.37	6.4 × 10^−13^
rs17652819	chr5:97,743,514	RP11-72K17.1	downstream	TAG.14.0.18.2.18.2	3.08	0.42	8.7 × 10^−13^
rs1156282	chr20:12,897,778	LINC01722	intron	TAG.15.0.16.0.18.1	1.18	0.16	1.0 × 10^−12^
rs13353012	chr1:55,190,415	USP24	intron	PE.42.7	1.53	0.21	1.2 × 10^−12^
rs964910	chr3:20,747,383	SGO1-AS1	intron	TAG.18.2.18.2.20.4	1.26	0.18	2.4 × 10^−12^
rs10042022	chr5:113,956	PLEKHG4B	intron	TAG.18.2.18.2.20.4	2.3	0.32	3.3 × 10^−12^
rs11978191	chr7:140,493,059	MKRN1	intergenic	TAG.14.0.18.2.18.2	2.82	0.4	4.0 × 10^−12^
rs13216190	chr6:36,737,907	RAB44	downstream	TAG.17.0.18.1.18.1	2.07	0.3	6.3 × 10^−12^
rs9505514	chr6:922,407	RP11-157J24.2	intergenic	PI.34.0	0.99	0.14	6.9 × 10^−12^
rs16876602	chr6:47,836,700	OPN5	downstream	TAG.18.2.18.2.18.2	1.34	0.19	8.2 × 10^−12^
rs17437994	chr14:30,774,693	RP11-159L20.2	intron	TAG.14.0.16.1.18.2	1.14	0.16	1.2 × 10^−11^
rs11989919	chr8:32,645,107	NRG1	intron	PC.30.2	0.91	0.13	1.2 × 10^−11^
rs4793823	chr17:56,662,843	NOG	intergenic	TAG.16.1.16.1.16.1	2.16	0.31	1.3 × 10^−11^
rs7915972	chr10:45,338,927	OR13A1	intergenic	TAG.18.2.18.2.20.4	1.3	0.19	1.5 × 10^−11^
rs13106855	chr4:47,411,505	GABRB1	intron	Glc.GalCer.d16.1.16.0	0.37	0.05	1.5 × 10^−11^
rs7827310	chr8:55,493,291	XKR4	intron	TAG.14.1.16.0.18.1	1.65	0.24	2.0 × 10^−11^
rs11071063	chr15:54,239,225	UNC13C	intron	TAG.14.0.18.2.18.2	2.46	0.36	2.9 × 10^−11^
rs11118256	chr1:206,492,504	IKBKE	intron	TAG.16.1.16.1.16.1	2.05	0.3	3.0 × 10^−11^
rs155594	chr2:157,413,630	CYTIP	downstream	TAG.18.2.18.2.18.2	2.12	0.32	5.0 × 10^−11^
rs1926868	chr1:183,986,440	COLGALT2	intron	PI.32.0	0.95	0.14	9.2 × 10^−11^

*Explanations of table columns* 3rd and 4th columns, genes and their genomic regions to which the SNPs map; 5th column, the lipid species to which the SNPs are associated with; 6th column, effect size of the SNPs on the lipid species; 7th column, standard error. *SE* Standard error.

The list of 34 newly identified independent short nucleotide polymorphisms (SNPs) associated with different lipid species in this study with study-wide statistical significance *p*-value < 1.1 × 10^−10^.

### Identification of lipidomic subgroups with distinct genetic component using PGMRA

The PGMRA analysis was done with the lipidome data and genetic data containing 18,370 unique SNPs preselected from GWAS*.* PGMRA identified 71 lipidome and 153 genotype biclusters. The detailed information about the genotype and lipidome biclusters (list of lipids or SNPs in each bicluster) has been presented in Supplementary Tables 4S and 5S. There were altogether 10,863 (153 genotype biclusters × 71 lipidome biclusters) candidates for lipidome-genotype relations analysis using hypergeometric statistics-based participants overlap test between each pair of biclusters from lipidome and genotype data. A total of 93 significant many-to-many lipidome-genotype relations were identified with hypergeometric *p*-value < 0.01 [Table 5S]. Genetic biclusters of the 93 lipidome-genotype relations contained 5,977 unique SNPs mapping to 3,164 different genes [Supplementary Table 6S]. Based on the SNPs and participants in the genetic biclusters of the 93 relations, we defined 29 of the relations with biclusters containing more than 50% unique SNPs and participants as the most distinct relations. The most distinct relations might represent different genetic-lipidomic subgroups among the studied population (Table 3). We pruned the SNPs in the genetic biclusters of the 29 most distinct relations based on LD to estimate the independent number of SNPs. The number of independent SNPs left in each of the biclusters from the 29 relations after SNP pruning is shown in column 4 of Table 3. Further, we compared the participants in each of the biclusters of the 29 relations with the rest of the participants with respect to total cholesterol (TC), LDL-cholesterol (LDL-C), HDL-cholesterol (HDL-C), triglycerides (TG), BMI, blood glucose level, blood insulin level and systolic and diastolic blood pressure using two-sample t-test.

**Table 3:** The 29 significant and most distinct (> 50% unique SNPs and participants) genotype-lipidome relations.

Relation ID	Number of shared participants	Number of SNPs	Number of independent SNPs	Number of lipid species	Hypergeometric *p*-value	Associated clinical variables (*p*-value)*
R9	62	1447	1191	17	5.1 × 10^−19^	TC (0.0002), Systolic BP (0.0001), diastolic BP (0.0005), LDL-C (0.002)
R7	29	1447	1191	14	1.2 × 10^−13^	TC (0.02), systolic BP (7 X 10^−06^), diastolic BP (0.0008), LDL-C (0.04), Blood glucose (0.02)
R5	46	1447	1191	22	4.0 × 10^−09^	TG (0.01), CRP (0.02), Systolic BP (0.0001), diastolic BP (2.4 X 10^−06^)
R68	27	27	17	82	3.1 × 10^−06^	TC (0.001), HDL C (0.0002), TG (0.002), diastolic BP (0.04), LDL-C (0.0006), blood insulin (0.03)
R1	25	1447	1191	11	8.6 × 10^−06^	HDL C (0.03)
R184	21	12	4	82	1.7 × 10^−05^	BMI (0.01), HDL-C (0.0003), TG (0.003), blood glucose (0.01), blood insulin (0.01)
R107	7	232	173	30	4.2 × 10^−05^	HDL-C (0.03), TG (0.03), systolic BP (0.002), diastolic BP (0.02)
R162	6	54	28	12	1.6 × 10^−04^	TC (4 X 10^−05^)
R119	9	34	19	30	4.6 × 10^−04^	HDL-C (0.001), TG (0.007), diastolic BP (0.05)
R103	5	193	123	28	4.7 × 10^−04^	TG (0.006)
R156	6	90	45	14	6.0 × 10^−04^	TG (0.005)
R101	11	8	2	82	6.2 × 10^−04^	TC (0.01), HDL-C (0.01), LDL-C (0.04)
R93	7	50	9	17	7.3 × 10^−04^	Systolic BP (0.05), diastolic BP (0.05), LDL-C (0.05)
R188	14	19	3	22	8.3 × 10^−04^	-
R57	9	19	7	30	9.9 × 10^−04^	TG (0.02)
R153	7	13	3	82	1.5 × 10^−03^	TC (0.008), HDL-C (0.04), LDL-C (0.004)
R94	5	7	3	23	1.7 × 10^−03^	TC (0.04), TG (0.05)
R32	15	65	24	82	2.1 × 10^−03^	TC (0.01), LDL-C (0.02), blood glucose (0.006), blood insulin (0.02)
R89	8	38	16	30	3.2 × 10^−03^	Systolic BP (0.003)
R10	16	1447	1191	10	3.6 × 10^−03^	systolic BP (0.05), diastolic BP (0.008), blood insulin (0.005)
R181	7	14	1	16	3.7 × 10^−03^	-
R45	11	114	62	16	4.0 × 10^−03^	HDL-C (0.003), diastolic BP (0.02)
R6	11	1447	1191	14	6.1 × 10^−03^	Systolic BP (0.02), diastolic BP (0.004)
R8	17	1447	1191	28	6.1 × 10^−03^	TC (0.03)
R58	5	19	7	29	6.6 × 10^−03^	TG (0.04), CRP (0.05), blood glucose (0.03)
R0	23	59	24	82	7.1 × 10^−03^	BMI (0.01), TC (0.008), HDL-C (0.0001), TG (3.8 X 10^−07^)
R3	22	1447	1191	19	7.9 × 10^−03^	TC (0.03), LDL-C (0.04)
R164	7	10	2	16	8.1 × 10^−03^	BMI (0.004)
R104	5	10	1	36	9.9 × 10^−03^	HDL-C (0.05), TG (0.02), blood insulin (0.009)

*Explanations of table columns* 2nd column, number of shared participants between genotype and lipidome biclusters; 3rd and 4th columns, number of SNPs in the genetic bicluster before and after linkage disequilibrium (LD)-based SNP pruning respectively; 5th column, number of lipid species in the lipid bicluster; 6th column, hypergeometric *p*-values for the overlap of participants in the genotype and lipidome biclusters of the relation; 7th column, clinical variables with respect to which the participants in the genetic biclusters of the corresponding genotype-lipidome relations are significantly (*p*-values in parentheses) different as compared to the rest of the participants assessed with *two-sample t-test. *TC* Total cholesterol; *HDL-C* high density lipoprotein cholesterol; *LDL-C* Low density lipoprotein cholesterol; *CRP* C-reactive protein; *TG* Triglycerides; *BP* Blood pressure.

The lipidomic biclusters in the most distinct genotype-lipidome relations were different from each other with respect to the classes of lipid species they were populated with. For example, while all the lipid species in the lipidome bicluster P20.18 belonged to class sphingolipid, biclusters P.15.7, P14.8 and P13.12 contained majority (> 75%) of lipid species belonging to the same class [Supplementary Table 4S]. Similarly, lipidome biclusters such as P13.11 and P10.3 contained more than 93% lipid species belonging to glycerophospholipid class [Supplementary Table 4S].

Among the 93 significant relations, there were 17 genotype biclusters, each of which were related to more than one lipidome biclusters. For example, in relations R66-R71, genotype bicluster G12.1 was associated with five different lipidome biclusters [Supplementary Table 7S]. Most of the lipid species in these lipidome biclusters belonged to sphingolipid and glycerophospholipid class. Difference among these lipidome biclusters were due to different molecular properties of the constituent lipid species. These observations uncover the complex genetic architecture of human plasma lipidome where the same genetic network may regulate multiple phenotypic outcomes (i.e., pleiotropy or multifinality). Similarly, there were 19 lipidome biclusters each of which were related to more than one genotype biclusters (i.e., equifinality), thereby uncovering the complex genotypic-phenotypic architecture of the lipidome.

### Biological pathways enriched in the SNPs of the most distinct lipidome-genotype relations

**Figure 4:**
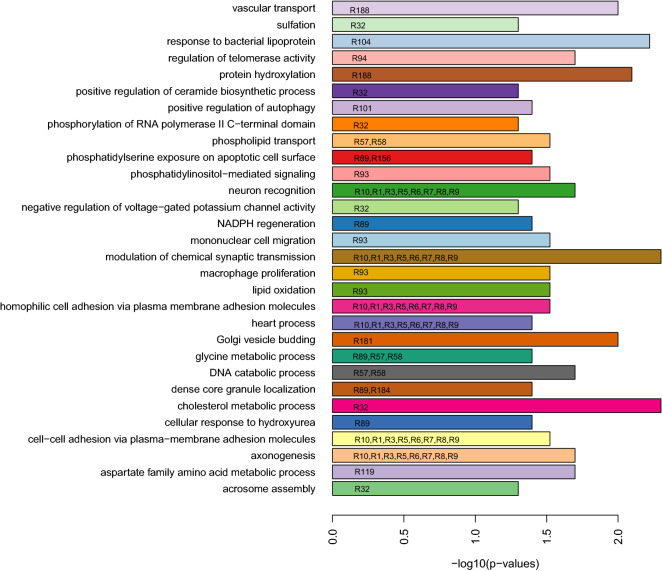
Biological processes (y-axis) from Gene Ontology database that were significantly (FDR < 0.05, x-axis) enriched in SNPs sets from 21 out of the 29 most distinct genotype-lipidome relations. Text within the bars represent the relation (R) identification numbers in which the corresponding biological processes were enriched.

The biological significance of the 29 most distinct genetic-lipidomic relations was analyzed by performing pathway enrichment analysis of the list of SNPs from the genetic biclusters of the the relations. We identified 30 gene ontology based biological processes significantly enriched in SNPs from 21 out of 29 most distinct genotype-lipidome subgroups (FDR < 0.05) (Fig. 4). Several biological processes among the list were related to lipid metabolism, inflammation process and immune system.

**Figure 5:**
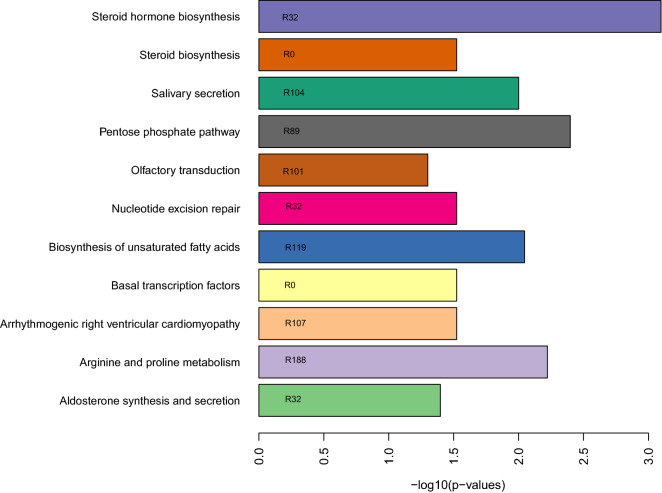
Biological pathways (y-axis) from Kyoto Encyclopedia of Genes and Genomes (KEGG) database that are significantly (FDR < 0.05, x-axis) enriched in SNPs sets from 8 out of the 29 most distinct genotype-lipidome relations. Text within the bars represent the relation (R) identification numbers in which the corresponding biological pathways were enriched.

Similarly, pathway enrichment analysis was also done with biological pathways from KEGG database. We identified 11 pathways enriched in eight out of the 29 distinct genotype-lipidome relations that included pathways related to lipid metabolism and heart disease (Fig. 5).

## Discussion

In this study, we implemented PGMRA, a novel machine learning approach to augment traditional GWAS of human plasma lipidome followed by pathway enrichment analysis to reveal the complex hidden genetics of human lipidome and its biological significance. Using traditional GWAS of 437 lipid species using genotyped variants, we replicated 21 SNPs and identified 34 new independent SNPs associated with different lipid species of human lipidome as compared to previous studies^21,23^. Our results suggest that human plasma lipidome from the participants of the YFS cohort has at least 29 genetically distinct subgroups and are influenced by genetic variations in genes related to biological processes such as lipid metabolism, inflammation process and immune system. The lipidomic biclusters of the distinct subgroups mostly contained lipid species belonging to classes sphingolipid and glycerophospholipid that are known to play crucial role in health and disease^41–43^.

The study identified several biological processes and pathways, including those related to lipid metabolism, significantly enriched in 21 of the 29 genetically most distinct lipidome subgroups. The significance of these biological processes in the molecular biology of atherosclerosis and in other lipid related metabolic and degenerative disease in humans remains largely uncertain and warrants further studies. Uncovering the hidden risk architecture of these subgroups of individuals with distinct genotypic and lipidomic profiles opens the opportunity to develop specific diagnostic tests as targets for precise clinical interventions. This opportunity will address the limitation of traditional evidence-based protocols when applied to complex phenotypes like atherosclerosis. Traditional evidence-based findings can only detect differences between the averages of heterogeneous groups and fail to indicate what is most effective in any particular individual.

While traditional GWAS can identify loci associated with a trait across the whole studied population, it disregards potential subgroups within the studied population and their associations with the studied trait. Consequently, subgroups' specific loci with smaller effect sizes are missed by tradition GWAS due to lack of sufficient statistical power. We speculate that the problem of missing heritability of a trait is perhaps due to failure of GWASs to identify complete genetic determinants of complex traits across population subgroups.

A recent GWAS study of 141 lipid species with ~ 9.3 million genetic variants in 2181 individuals reported 35 lipid-species-associated loci with *p*-value < 5 × 10^−8^^21^. In comparison, the present study identified 5,977 unique SNPs across 93 sub-populations represented by the 93 genotype-lipidome relations identified by PGMRA. The 5,977 SNPs map to 3,164 different genes replicating 13 of the genes reported by^21^ and extending the current knowledge by a total 3129 novel lipidome associated genes. The differences between the studies come from the substantially wider LC–MS/MS based analysis platform of 437 lipids and alternative machine learning approach used in this study as compared to only 141 lipid species used by^21^ for traditional GWAS. The most recent trans-ancestry meta-analyses in 1.65 million individuals including 350,000 non-Europeans identified 941 clinical lipid-associated loci including 355 new loci from either single- or multi-ancestry analyses^10^. From these novel findings, three of the reported SNPs were replicated in the present study with a substantially lower number of subjects (~ N = 1500). The second largest GWAS study of four clinical lipid traits (HDL-C, LDL-C, total cholesterol and triglycerides) with ~ 600 000 participants and 32 million genetic markers identified 826 independently associated lipid variants with genome-level significance (*p*-value < 5.0 × 10^−8^)^9^. The 826 lipid variants contained 118 novel loci and 268 previously identified loci^8,9^. The present study replicated 78 of the 386 loci with substantially smaller sample size (~ N = 1500 vs. ~ 600.000), highlighting the importance of our novel GWAS-PGMRA approach.

Early prediction of risk of CVDs is a cornerstone of disease prevention and could greatly reduce the enormous socio-economic burden posed by CVDs^44^. The PGMRA approach identifies genetic-lipidomic subgroups within the study population allowing a gene-based classification of plasma lipidome. The distinct genetic variants in the subgroups may contribute synergistically or additively to the risk of dyslipidemias and may be useful to develop precision diagnostics and prognostics for lipid related cardiometabolic as well as other degenerative diseases. Previously, the PGMRA approach has discovered genetic subgroups of schizophrenia associated with distinct gene products and clinical syndromes^45^. The genetic information of subgroups can potentially be used for risk prediction and stratification already in very young age as genetic risks persist starting from fetal period.

This study has several limitations. A major limitation is the lack of validation of the results in an independent multi-ethnic population-based cohorts. Validation or replication of the results requires availability of both genetic and lipidomic data from comparable platforms which was unavailable to our knowledge during the period of this study. Our previous studies with complex traits of temperament, character and personality, however, suggests that reliability of PGMRA method as the results from Finnish population were highly replicable (80 to 90%) in independent data from Germans and Koreans cohorts^26,27^. Another limitation is that the study is based on genotype data without imputation and therefore may have missed many genetic variants. We chose to focus on accurate, non-imputed and hence smaller data to showcase the implication of the proposed alternative machine-learning approach to analyze the complex genetics of human plasma lipidome. Given the promising results in the current study, similar analysis with imputed genotype data from multiple cohorts is warranted. Accurate identification of causal SNPs of complex traits among candidate set of SNPs in LD based purely on statistical evidence is difficult. Because of this, LD-based pruning of SNPs does not guarantee to retain the causal SNPs for further analysis. That is why, it is not advisable to perform LD-based pruning of SNPs before biclustering or any similar analysis to prevent the loss of (genetic) information. Because of these reasons, we did not perform LD-based pruning of SNPs before PGMRA in this study. As a consequence, we acknowledge that the identified biclusters may contain SNPs in LD with some SNPs that do not have true association with the studied traits. While we reported the number of independent SNPs obtained by LD-based pruning of SNPs in each of the biclusters obtained using PGMRA, this issue however requires consideration in further analyses of the SNP sets, such as in association analysis of the SNP sets or genetic risk scores (GRS) calculated using the SNP sets with phenotypes of interest. For example, LD pruning or clumping of the SNPs in a bicluster should be performed before calculation of GRS. A study by^46^ suggests that modelling the LD structure rather than filtering out SNPs based on a LD threshold improves prediction accuracy of GRS by reducing information loss.

## Conclusion

The study identified 29 distinct genotype-lipidome subgroups in the YFS participants that are influenced by genetic variations in genes related to biological processes such as lipid metabolism, inflammation process and immune system. The study presents an alternative ML-based research methodology in the field of genetics and lipidomics that provides potentially a ground-breaking resolution for the missing heritability problem for cardiovascular or any other lipid related diseases. The study proposes a step towards the direction of new genetic-based classification of polygenic dyslipidemias and their implication in early risk stratification for cardiovascular or other lipid related diseases and stimulates additional studies in the field of personalized and predictive medicine for CVDs. In addition, the study showcases a ML approach for multiomics integration that can be applied to other biomedical domains.

## Supplementary Information

Supplementary Information 1.

Supplementary Information 2.

## Data Availability

The YFS dataset comprises health related participant data and their use is therefore restricted under the regulations on professional secrecy (Act on the Openness of Government Activities, 612/1999) and on sensitive personal data (Personal Data Act, 523/1999, implementing the EU data protection directive 95/46/EC). Due to these legal restrictions, the Ethics Committee of the Hospital District of Southwest Finland has in 2016 stated that individual level data cannot be stored in public repositories or otherwise made publicly available. Data sharing outside the group is done in collaboration with YFS group and requires a data-sharing agreement with the understanding that collaborators will protect the data and not share it with any other parties. The list of all investigators that collaborate with the YFS group is displayed at the website of the YFS (http://youngfinnsstudy.utu.fi/). Investigators can submit an expression of interest to the chairman of the data sharing and publication committee (prof Mika Kähönen, Tampere University) in the case of clinical data and in genomic data to professor Terho Lehtimäki (Tampere University).
