# Supplementary material for: Uncovering the complex genetic architecture of human plasma lipidome using machine learning methods
Source: Sci Rep. 2023 Feb 22;13:3078. doi: 10.1038/s41598-023-30168-z (PMC9947228; doi:10.1038/s41598-023-30168-z)
Supplement: Supplementary file 2 — Supplementary Information 2. [file 41598_2023_30168_MOESM2_ESM.pdf]

Uncovering the complex genetic architecture of human plasma lipidome using machine learning methods

Supplementary Figures

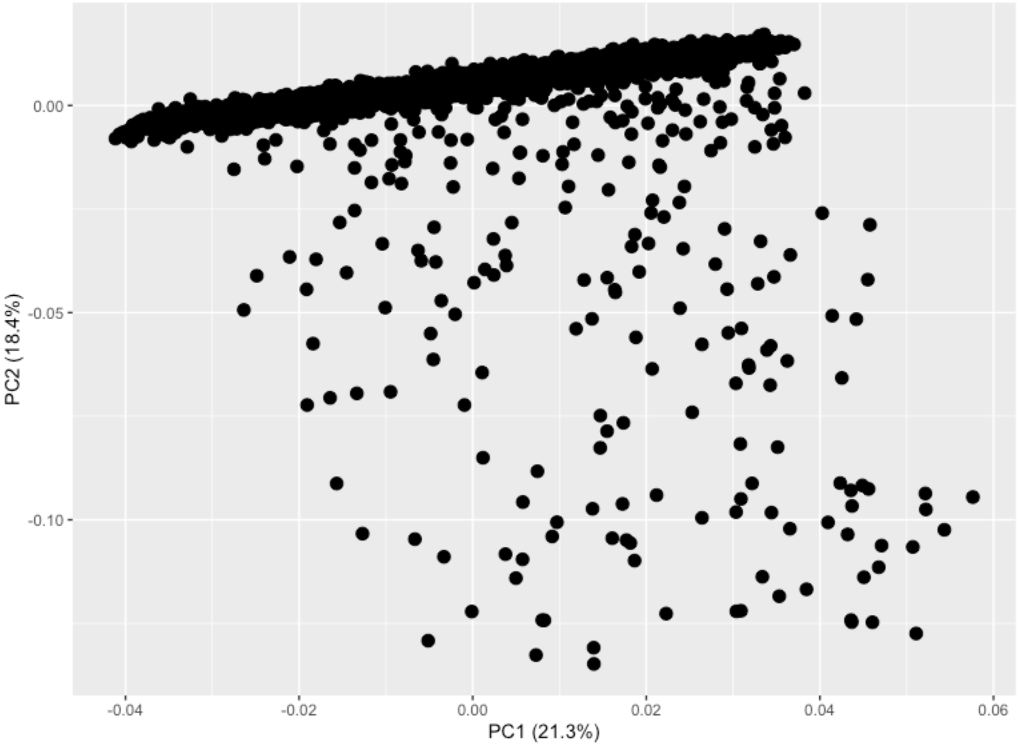

Figure S1: Genetic data (genotyped only) based principal component analysis (PCA) plot illustrating genetic relatedness of the Young Finns Study (YFS) participants (n=1426) of this study.

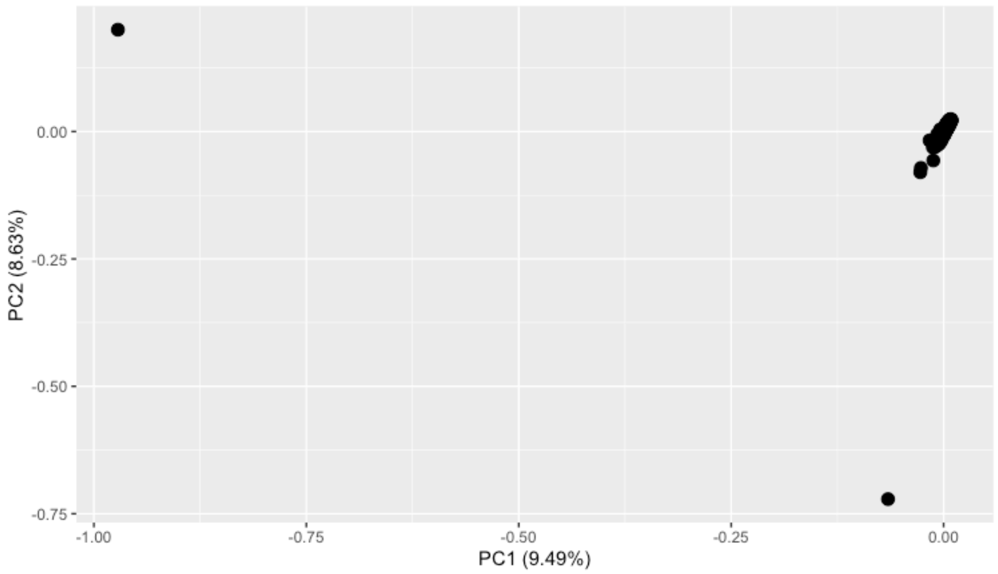

Figure S2: Imputed genetic data (using TOPMed Version R2 reference panel) based principal component analysis (PCA) plot illustrating genetic relatedness of the Young Finns Study (YFS) participants (n=1426) of this study.
